# The *Orthotospovirus* nonstructural protein NSs suppresses plant MYC-regulated jasmonate signaling leading to enhanced vector attraction and performance

**DOI:** 10.1371/journal.ppat.1007897

**Published:** 2019-06-17

**Authors:** Xiujuan Wu, Shuang Xu, Pingzhi Zhao, Xuan Zhang, Xiangmei Yao, Yanwei Sun, Rongxiang Fang, Jian Ye

**Affiliations:** 1 State Key Laboratory of Plant Genomics, Institute of Microbiology, Chinese Academy of Sciences, Beijing, China; 2 University of the Chinese Academy of Sciences, Beijing, China; Eidgenossische Technische Hochschule Zurich, SWITZERLAND

## Abstract

Pandemics of vector-borne human and plant diseases often depend on the behaviors of their arthropod vectors. Arboviruses, including many bunyaviruses, manipulate vector behavior to accelerate their own transmission to vertebrates, birds, insects, and plants. However, the molecular mechanism underlying this manipulation remains elusive. Here, we report that the non-structural protein NSs of Tomato spotted wilt orthotospovirus, a prototype of the *Tospoviridae* family and the *Orthotospovirus* genus, is a key viral factor that indirectly modifies vector preference and increases vector performance. NSs suppresses the biosynthesis of plant volatile monoterpenes, which serve as repellents of the vector western flower thrips (WFT, *Frankliniella occidentalis*). NSs directly interacts with MYC2, the jasmonate (JA) signaling master regulator and its two close homologs MYC3 and MYC4, to disable JA-mediated activation of *terpene synthase* genes. The dysfunction of the MYCs subsequently attenuates host defenses, increases the attraction of thrips, and improves thrips fitness. Moreover, MYC2 associated with NSs of Tomato zonate spot orthotospovirus, another Euro/Asian-type orthotospovirus, suggesting that MYC2 is an evolutionarily conserved target of *Orthotospovirus* species for suppression of terpene-based resistance to promote vector performance. These findings elucidate the molecular mechanism through which an orthotospovirus indirectly manipulates vector behaviors and therefore facilitates pathogen transmission. Our results provide insights into the molecular mechanisms by which *Orthotospovirus* NSs counteracts plant immunity for pathogen transmission.

## Author summary

Most bunyaviruses are transmitted by arthropod vectors, and some of them can modify the behaviors of their arthropod vectors to increase transmission to mammals, birds, and plants. NSs is a non-structural bunyavirus protein with multiple functions that acts as an avirulence determinant and silencing suppressor. In this study, we identified a new function of NSs as a conserved manipulator of vector behavior via plant. NSs suppresses jasmonate-mediated plant immunity against thrips by directly interacting with several homologs of MYC transcription factors, the core regulators of the jasmonate-signaling pathway. This hijacking by NSs enhances thrips preference and performance. Therefore, our data support the hypothesis that MYC2 is a convergent target that plant pathogens manipulate to promote their survival in plants.

## Introduction

Arthropod-borne viruses (arboviruses) are virulent causal agents of diseases in humans, animals, and plants. Vector behaviors have critical ecological and evolutionary consequences for arboviruses, which rely exclusively on their arthropod vectors for dispersal to new hosts. Therefore, it is of evolutionary significance for an arbovirus to alter its vector’s behavior to facilitate its own transmission. For plant viruses, such influence of vectors by viruses can include plant-mediated indirect effects or direct manipulation within the vector after acquisition. Among the indirect effects, infected plants tend to be more attractive to vectors [1]. For example, *Geminiviridae* and *Luteoviridae* viruses almost universally induce preferred settling of the vectors onto infected plants [2–5], and this phenomenon also exists among the *Potyviridae* and *Bunyaviridae* [6–9]. Moreover, viruses can positively or negatively affect the performance or fitness of arthropod vectors on the host. Persistently transmitted viruses, which need a sustained feeding of insect vectors to be acquired or transmitted, in particular, have positive effects on vector performance. For example, insect vectors perform better on *Geminiviridae-* and *Tospoviridae*-infected plants [9–12]. For nonpersistently transmitted viruses, vectors acquire or transmit the viruses in seconds through probing or feeding, such as *Potyviridae*, *Caulimoviridae* and *Bromoviridae*, also can positively or negatively affect their vectors for efficient virus spread [1, 6, 13–15].

*Bunyavirales* encompasses nine families of viruses with single-stranded negative-sense RNA genomes. As a prototype of the plant-infected *Tospoviridae* family, Tomato spotted wilt orthotospovirus (TSWV) is transmitted mainly by *Frankliniella occidentalis* Pergande (Western flower thrips, WFT) in a persistent and propagative manner [16,17]. Plant infection with TSWV influences several vector behaviors, such as biting and host choice to increase virus transmission, similar to the animal-infecting members of *Bunyavirales* [18–20]. For instance, non-viruliferous *F*. *occidentalis* prefers to settle on TSWV-infected pepper (*Capsicum annuum* L.) and *Datura stramonium* plants over noninfected controls [9]. However, the underlying molecular mechanism of this conserved indirect manipulation of vector behaviors by *Orthotospovirus* and *Bunyavirales* species is still unclear, although this plant immunity suppression is thought to occur in TSWV-infected *Arabidopsis thaliana* [21]. The bunyavirus families are divided based on their different coding strategies for the additional non-structural proteins, NSm and NSs, which are often involved in host-pathogen interactions. *Orthotospovirus* NSm protein facilitates the movement of viral ribonucleoproteins from cell to cell within the plant host. NSm of TSWV has recently been identified as the avirulence factor recognized by the product of resistance gene *Sw-5b* from tomato (*Solanum lycopersicum* L.) [22]. The NSs proteins of many bunyaviruses modulate host innate immune responses, and NSs in *Orthotospovirus* functions as a silencing suppressor in both plants and insects [23,24]. These proteins are responsible for establishing systemic infection in plants and for virus transmission by insect vectors [25,26].

Many plant species emit herbivore-induced plant volatiles (HIPVs), as an indirect anti-herbivore defense strategy [27–30]. HIPVs can repel insects such as aphids and caterpillars or deter lepidopteran oviposition [31–33], and are a common induced defense mechanism among plants including cotton and tomato [34,35]. Phytohormones such as jasmonate (JA) play vital roles in regulating HIPV production upon insect attack [36,37]. Several viruses have been shown to modify this JA-regulated volatile biosynthesis to affect the communication between plant and insect vector. For instance, begomoviruses inhibit the JA pathway and modify volatile terpene-mediated defense responses against whitefly [38]. The JA-mediated biosynthesis of secondary metabolites is believed to be associated with thrips resistance [39]. However, whether and how TSWV influence JA signaling remains elusive, although this virus is thought to hijack the antagonistic relation between JA and salicylic acid signaling [40].

In this study, we showed that TSWV benefits to thrips vector by suppressing a JA-regulated defense pathway of plants against herbivores. We identified the NSs protein from thrip-borne TSWV as a viral genetic factor induced attraction of its insect vector. Various NSs from *orthotospovirus* suppress the JA signaling pathway in the host plant by directly interacting with MYCs, key regulators of the JA signaling pathway, to reduce host defense responses against thrips. Our results establish a molecular mechanism underlying how TSWV attracts and benefits to its thrips vector by targeting plant MYC proteins.

## Results

### TSWV infection enhances plant attractiveness to the thrips vector and suppresses plant terpene synthesis

**Fig 1:**
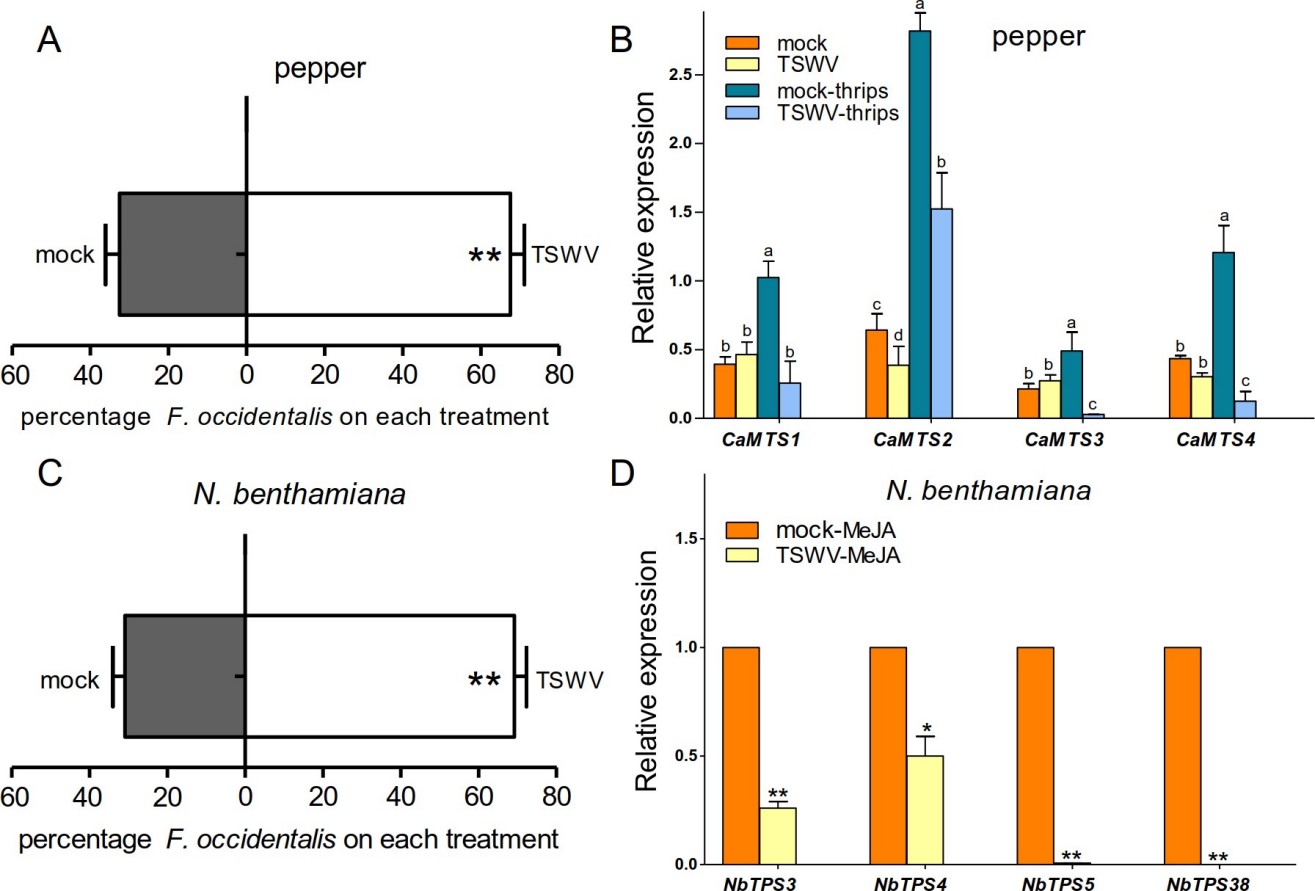
TSWV infection enhances plant attractiveness to the thrips vector and suppresses plant terpene synthesis. (A) Thrips preference (as percentage recaptured WFT out of 50 released) on different pepper plants. Four-week-old pepper plants were infected with TSWV (TSWV) or inoculated with buffer (mock). Plants of a similar size were used for thrips bioassay at 14 days post viral infection (dpi). Data are mean + SE, n = 6. **P < 0.01, Wilcoxon matched pairs tests. (B) Relative expression levels of *TPS* genes in pepper with or without thrips infestation for 6 h. Values are means + SE, n = 3. *P* < 0.05, one-way ANOVA plus Duncan’s multiple range tests. (C) Thrips preference (as percentage recaptured WFT out of 50 released) on *N*. *benthamiana* plants. Three-week-old *N*. *benthamiana* plants were infected with TSWV (TSWV) or inoculated with buffer (mock). Leaves of a similar size were used for the thrips bioassay at 14 dpi. Data are mean + SE, n = 6. **P < 0.01, Wilcoxon matched pairs tests. (D) Relative expression levels of various *TPS* genes in mock or TSWV-infected *N*. *benthamiana* after MeJA treatment. *N*. *benthamiana* plants were sprayed with 100 μM MeJA (Sigma-Aldrich) containing 0.01% (v/v) Tween 20. Values are means + SE, n = 3. *P < 0.05 **P < 0.01, Student’s *t*-test.

We first investigated the indirect effect of TSWV infection on the behavioral responses of the vector *Frankliniella occidentalis* Pergande (Western flower thrips, WFT). We conducted a two-choice assay between infected and non-infected plants. Pepper (*Capsicum annuum* L.), a natural host of TSWV and an important crop worldwide, was first tested in the tripartite thrip–orthotospovirus–plant interaction. A group of 50 non-viruliferous WFT was released from the center of the two-choice arena between two types of pepper plants. Consistent with previous results from Maris et al. [9], ~68% of thrips approached TSWV-infected plants, whereas the remaining approached non-infected plants (Fig 1A), suggesting that TSWV infection indirectly increases the attractiveness of peppers to the thrips vector.

The attraction of insect vectors induced by the infection of other viruses is dependent on plant volatiles [38,41]. We therefore measured the expression levels of *terpene synthase* (*TPS*) genes in pepper leaves based on our previous functional analysis of *TPS* genes [38]. Reverse-transcription quantitative PCR (RT-qPCR) analysis showed that the expression of four pepper *monoterpene synthase* genes (*CaMTS1*, *CaMTS2*, *CaMTS3*, and *CaMTS4*), which are related to monoterpene synthesis, were upregulated after thrips infestation (Fig 1B). However, the terpene biosynthesis gene expression activated by thrips was significantly lower in TSWV-infected plants compared with the control (Fig 1B).

Another model (host) plant for tripartite interaction research, *Nicotiana benthamiana*, was also tested. Similar to the observations in pepper, TSWV-infected *N*. *benthamiana* leaves also were more attractive to thrips than non-infected leaves (Fig 1C). Moreover, RT-qPCR analysis indicated that the *terpene synthase* genes *NbTPS5* and *NbTPS38* responded to thrips infestation in *N*. *benthamiana* (S1A Fig). Consistent with the above results, *NbTPS5* and *NbTPS38* expression was notably induced by methyl jasmonate (MeJA) treatment, reflecting the same trends as during thrips infestation (S1B Fig). JA signaling is normally rapidly activated by thrips feeding [40]. Considering that *N*. *benthamiana* is not a good host for thrips as indicated by their poor survival rate, and the finding that MeJA induces similar expression of *TPS* genes in *N*. *benthamiana* as thrips infestation (S1 Fig), we used MeJA to mimic WFT infestation in further tripartite interaction experiments. The expression of *NbTPS3*, *NbTPS4*, *NbTPS5*, and *NbTPS38* was less changed in TSWV-infected plants compared to the control plants when induced by methyl jasmonate (MeJA) (Fig 1D).

### TSWV infection induces a terpene-dependent preference in the thrips vector

**Fig 2:**
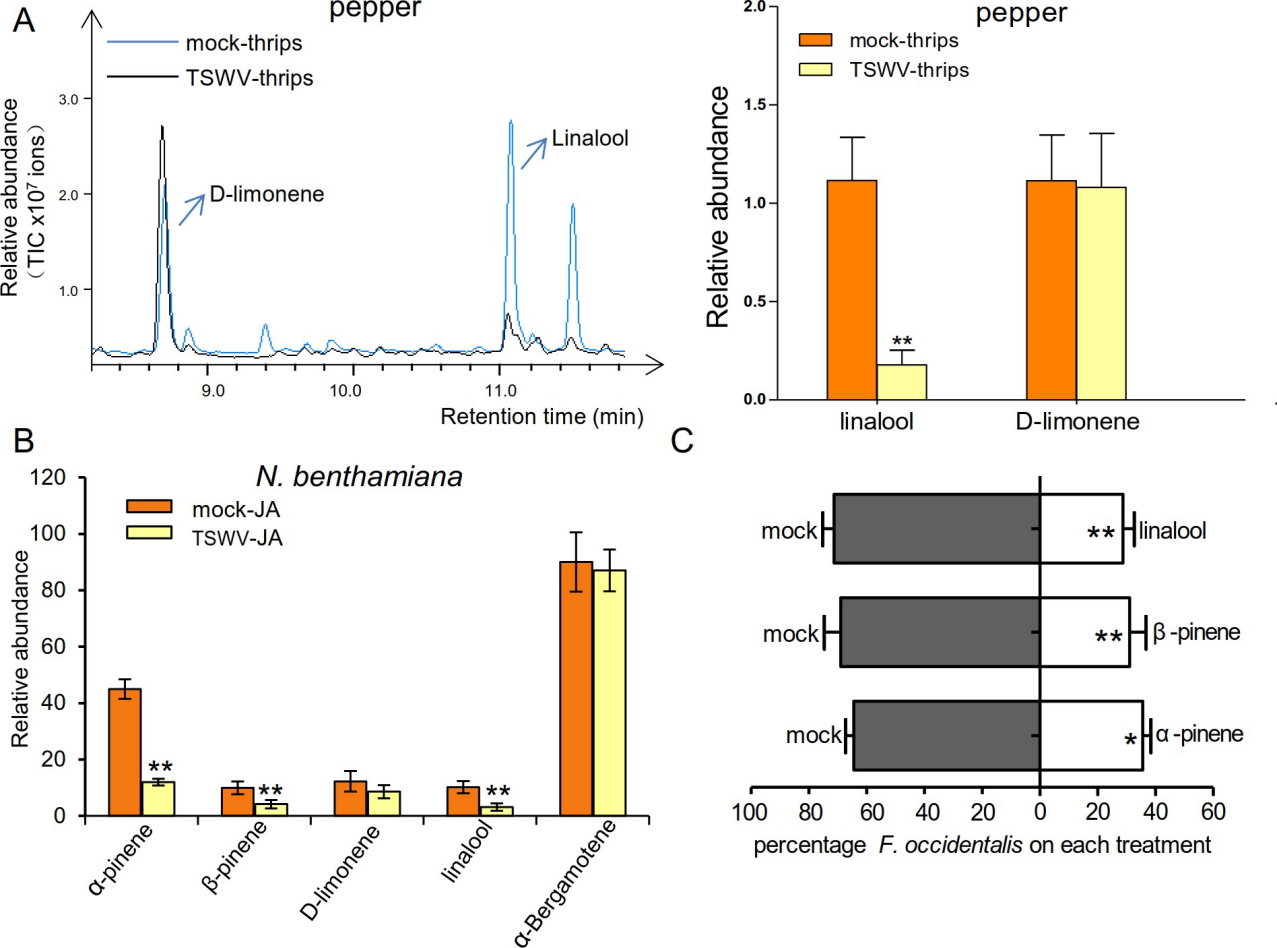
TSWV infection increases attractiveness to the thrips vector in a terpene-dependent manner. (A) Representative GC/MS ion chromatograms of the headspace volatile compounds of control (mock-thrips) and TSWV-infected peppers (TSWV-thrips) after thrips infestation for 6 h. The peaks of specific products are marked with arrows in the left panel. Relative abundance of terpenes emitted after thrips infestation are showed in the right panel. Values are means + SE, n = 4. **P < 0.01, Student’s *t*-test. (B) Terpenes emitted by *N*. *benthamiana* after TSWV infection (under MeJA treatment). Values are mean relative amounts (percentage of internal standard peak area) ± SE, n = 4. **P < 0.01, Student’s *t*-test. (C) Thrips preference (as percentage recaptured WFT out of 50 released) on the pure monoterpenes (linalool, α-pinene, β-pinene) and solvent control (n-hexane) in a two-choice assay. Data are mean percentages + SE, n = 6. *P < 0.05 **P < 0.01, Wilcoxon matched pairs tests.

To explore the metabolic consequences of the altered *TPS* gene expression, we investigated changes in the emission of plant volatile compounds after TSWV infection. Plants have evolved a blend of HIPVs that are emitted in response to, and directly repel, herbivores [27–33]. We measured the volatile emission collected in the headspace of peppers with or without thrips infestation. When infested by thrips, damaged plants emitted more volatiles than control plants (S2 Fig). It is noteworthy that TSWV-infected plants emitted significantly less linalool, which is the main monoterpene collected from peppers after herbivory, compared to non-infected plants, consistent with their lower expression of *TPS* genes. In addition, there was no significant difference in the emissions of the monoterpene D-limonene (Fig 2A). We also monitored the emission of volatile compounds in the headspace of *N*. *benthamiana*, after applying MeJA to mimic WFT infestation; this plant hormone is known to elicit the production of various terpenes [42]. Among the five detected terpenes, the levels of three volatile monoterpenes, linalool, α-pinene, and β-pinene, were significantly lower in TSWV-infected plants compared to non-infected plants (Fig 2B). To examine whether linalool, α-pinene and β-pinene play a role in plant–WFT interactions, we performed a two-choice assay in which non-viruliferous WFT had the choice between the changed monoterpenes and the solvent control hexane. The α-pinene and β-pinene directly repelled thrips similarly to linalool (Fig 2C). These consistent results on pepper and *N*. *benthamiana* revealed that TSWV infection induces a terpene-dependent preference in the thrips vector and that this feature is common among various TSWV hosts.

### NSs manipulates the preference behavior of WFT on plants

**Fig 3:**
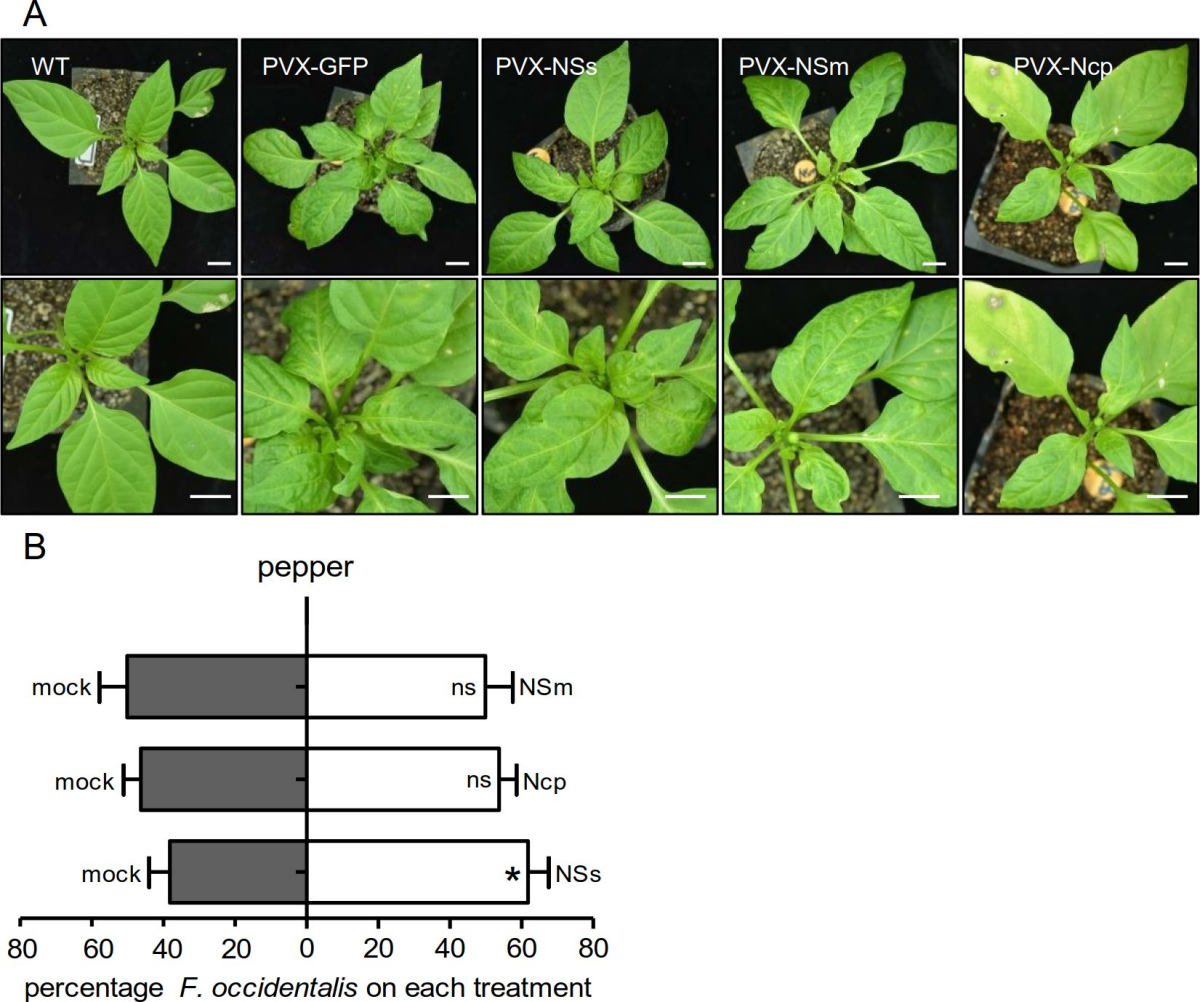
NSs from TSWV is a vector behavior manipulator. (A) Phenotype of pepper leaves inoculated with recombinant Potato virus X (PVX) vectors. PVX-NSs, PVX-NSm or PVX-Ncp was transformed into peppers via agroinfiltration. PVX-GFP was used as the control. Bar = 2 cm. (B) Attractiveness of different infiltrated peppers. Agrobacteria carrying individual recombinant PVX vectors were infiltrated into peppers. Plants of a similar size were used for thrips two-choice assays at 10 dpi. Data are mean choice percentages + SE, n = 6. *P < 0.05, Wilcoxon matched pairs tests.

Our data demonstrated that the orthotospovirus TSWV increases the attraction of insect vector WFT to its host plant by inhibiting *terpene synthase* expression in the host. Next, to explore which viral protein(s) in TSWV manipulate vector host choice, we selected three of the five viral proteins in TSWV, including a structural protein nucleocapsid protein (Ncp) and two non-structural proteins, NSm and NSs [24]. We used the heterologous Potato virus X (PVX) model system for systemic ectopic expression of individual genes for TSWV NSs, NSm or Ncp [43]. PVX-GFP, used to express green fluorescent protein (GFP) in the plant, was served as the control. There were no obvious morphological differences between these recombinant PVX vector-infected peppers (Fig 3A). We performed a WFT two-choice assay to determine whether the expression of a single viral protein is sufficient to attract WFT. PVX-NSs-infected plants but not PVX-NSm- or PVX-Ncp-infected plants were significantly more attractive to WFT than PVX-GFP-infected plants (Fig 3B), indicating the expression of *NSs* alone is sufficient to attract WFT in peppers.

### TSWV NSs interacts with MYC2 and its homologs MYC3 and MYC4

**Fig 4:**
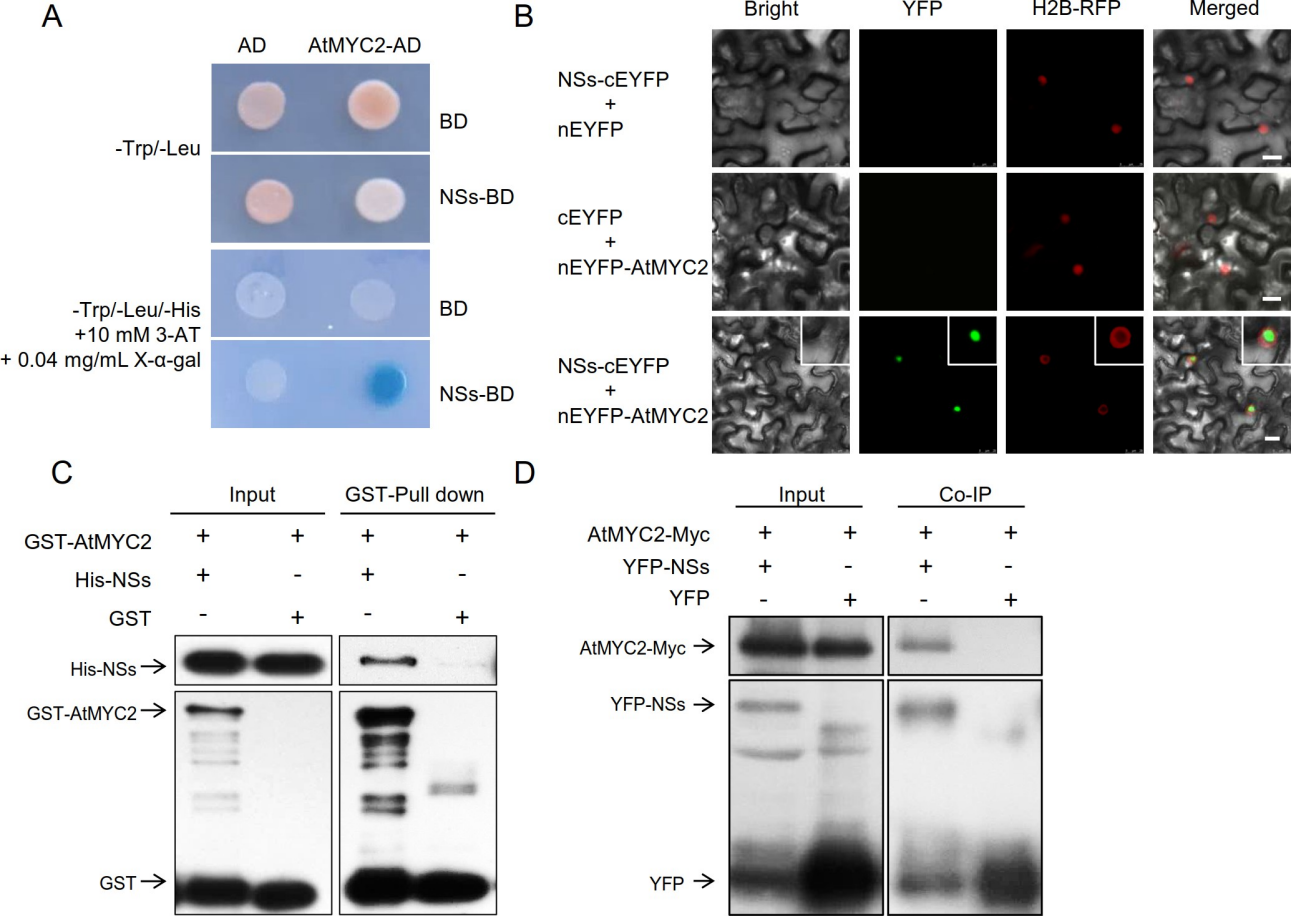
TSWV NSs interacts with MYC2. (A) Yeast two-hybrid assay between NSs and AtMYC2. Yeast cotransformed with the indicated plasmids was spotted onto synthetic medium (SD-Leu-Trp-His) containing 0.04 mg/mL X-α-gal and 10 mM 3-amino-1,2,4-triazole (3-AT). The empty vectors pGBKT7 (BD) and pGADT7 (AD) were used as negative controls. (B) Bimolecular fluorescence complementation (BiFC) assay. NSs-cEYFP and nEYFP-AtMYC2 were transiently expressed in H2B-RFP transgenic *N*. *benthamiana* leaf epidermal cells via agroinfiltration. Bars = 15 μm. (C) GST pull-down assays between NSs and AtMYC2. (D) Interaction between NSs and AtMYC2 in Co-immunoprecipitation (Co-IP) assay. Total protein was extracted from *N*. *benthamiana* leaves transiently expressing *35S*:*MYC2-Myc* together with *35S*:*YFP-NSs* or *35S*:*YFP* alone. GFP-trap beads were used to precipitate the interaction complex, Anti-GFP and Anti-Myc antibodies were using to detect the immunoprecipitates.

To explore the host protein targets of NSs, we screened an *Arabidopsis* cDNA library by yeast two-hybrid analysis and identified AtMYC2, a key components of the JA signaling pathway [44–46]. Based on the importance of the JA signaling pathway to plant–herbivore interactions, we further confirmed the interaction between AtMYC2 and NSs. In a yeast two-hybrid assay, the yeast transformants harboring AD-AtMYC2 and BD-NSs could grow on SD-Leu-Trp-His medium with 0.04 mg/mL X-α-gal and turned blue, while the negative control transformants did not (Fig 4A). A bimolecular fluorescence complementation (BiFC) assay confirmed the AtMYC2 and NSs interaction in plants. NSs-cEYFP and nEYFP-AtMYC2 constructs were co-expressed in transgenic *N*. *benthamiana* lines that expressed a nucleus-localized histone H2B-red fluorescent protein (H2B-RFP) fusion marker protein. A strong interaction (represented by fluorescence) was observed in the nucleus (Fig 4B), while no fluorescence was observed in the negative controls (Fig 4B). GST pull-down assay was used to verify the direct physical interaction between NSs and AtMYC2 *in vitro*. His-NSs was pulled down by GST-AtMYC2, but not by GST alone (Fig 4C). Moreover, in a co-immunoprecipitation (Co-IP) assay, AtMYC2-Myc was coimmunoprecipitated by YFP-NSs, but not the YFP control (Fig 4D). Taken together, these results demonstrate that NSs directly interacts with MYC2 *in vitro* and *in vivo*.

MYC3 and MYC4 are two closely related bHLH transcription factors that function partially redundantly with MYC2 to activate JA responses in *Arabidopsis* [47]. To determine whether TSWV NSs targets MCY3 and MYC4 as well, we performed a yeast two-hybrid assay and a BiFC assay. MYC2 relatives MYC3 and MYC4 were also found to interact with TSWV NSs as indicated by AD-AtMYCs (MYC3 and MYC4) and BD-NSs yeast transformants turned blue when grown on SD-Leu-Trp-His medium with 0.04 mg/mL X-α-gal (S3A Fig). In the BiFC assay, *N*. *benthamiana* coexpressing MYC3 and NSs exhibited fluorescence in the cytoplasm and nucleus, while coexpression of MYC4 and NSs led to fluorescence only in the cytoplasm (S3B Fig). These results indicate that MYC family transcription factors are targeted by NSs protein.

### MYCs positively regulate volatile-dependent immunity against WFT in *Arabidopsis*

**Fig 5:**
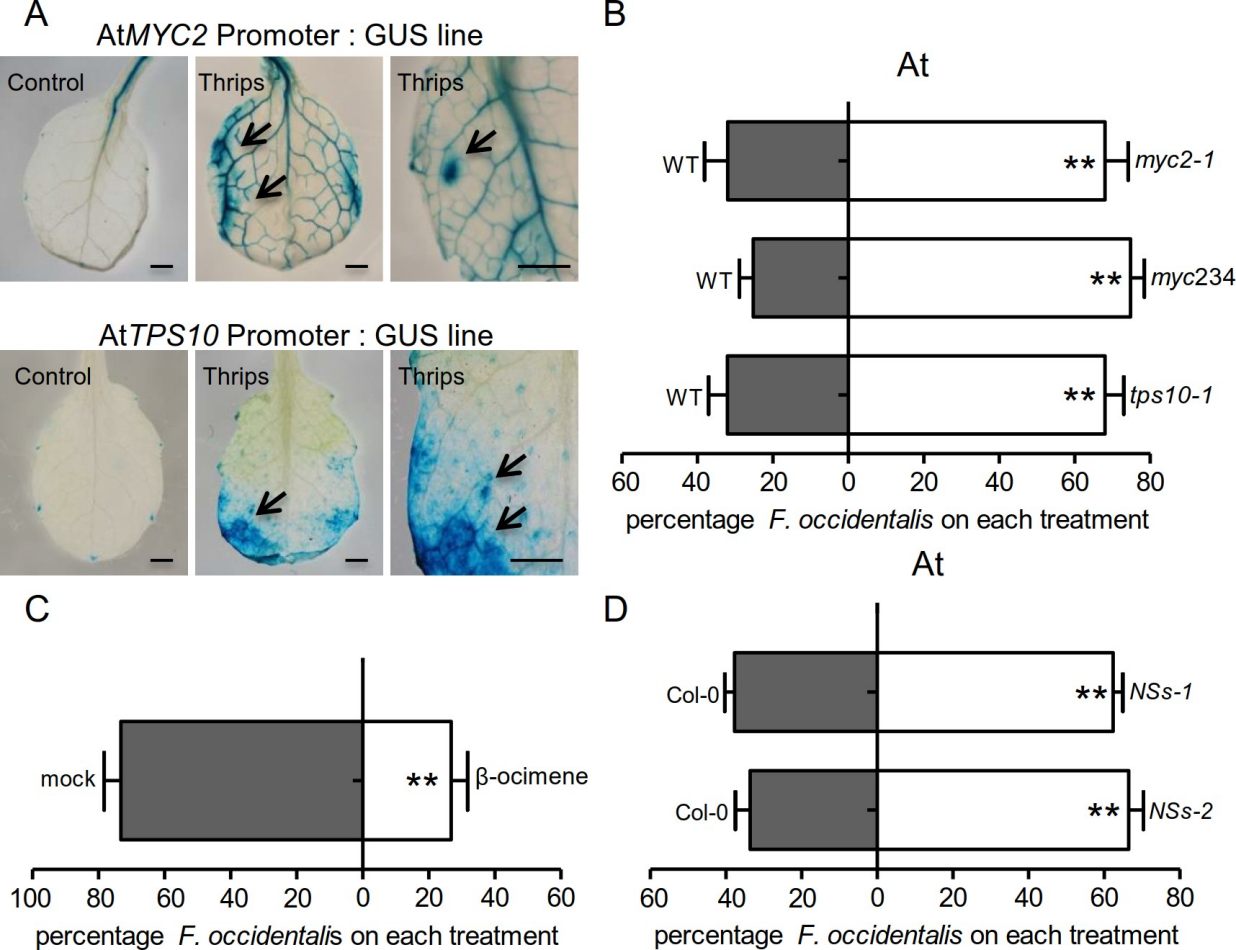
MYC2 and its homologs in *Arabidopsis* are essential regulators of host immunity responses against WFT. (A) GUS staining of *AtMYC2p-GUS* and *AtTPS10p*-*GUS* seedlings after 24 h of thrips feeding. An untreated line was used as a control. Arrows indicate thrips feeding sites. Bars = 2 mm. (B) Thrips preference (as percentage recaptured WFT out of 50 released) between the mutants and WT control in a two-choice assay. Three-week-old *Arabidopsis* plants cultured in MS medium were used for the thrips two-choice assay. Data are mean percentages + SE, n = 6. **P < 0.01, Wilcoxon matched pairs tests. (C) β-ocimene is less attractive to thrips than mock treatment in a two-choice assay. Data are mean percentages + SE, n = 6. **P < 0.01, Wilcoxon matched pairs tests. (D) Thrips preference (as percentage recaptured WFT out of 50 released) between the *35S*:*YFP-NSs* transgenic *Arabidopsis* lines (*NSs-1*; *NSs-2*) and mock control in a two-choice assay. Three-week-old transgenic *Arabidopsis* plants cultured in MS medium were used. Data are mean percentages + SE, n = 6. **P < 0.01, Wilcoxon matched pairs tests.

We previously showed that *Arabidopsis* MYC2 plays important roles in JA-regulated plant defense responses, e.g. directly regulates *TPS10* transcript levels to promote plant volatile biosynthesis [38]. Thus, we hypothesized that AtMYC2, which interacts with virulence factor NSs, is involved in the viral-induced, volatile-dependent attraction of WFT to the host plant. To validate this hypothesis, we performed a GUS staining assay using two transgenic *Arabidopsis* lines expressing an *AtMYC2* or *AtTPS10* promoter: *GUS* reporter gene. As shown in Fig 5A, high *GUS* expression was detected after 24 h of WFT infestation. This expression pattern suggests that *AtMYC2* and *AtTPS10* both function in defense responses against WFT in *Arabidopsis*.

To analyze the effects of *AtMYC2* and *AtTPS10* on the feeding preferences of thrips, we performed two-choice assays using *myc2-1*, *tps10-1*, and wild-type Col-0 *Arabidopsis*. As shown in Fig 5B, the *myc2-1* and *tps10-1* mutants were more attractive to WFT than wild type. We also tested the effect of triple mutant *myc234* on host preference, finding that WFT strongly preferred *myc234* plants over the wild type (Fig 5B). *AtTPS10* encodes a monoterpene synthase that produces β-ocimene [48]. We therefore carried out a two-choice assay of β-ocimene to examine whether the attraction of *tps10* is terpene-dependent. β-ocimene had a strong repellent effect on WFT (Fig 5C). These results indicate that *AtMYC2* is essential for terpene-dependent immunity against the thrips vector. We further examined if the TSWV NSs contributes to the preference of thrips on *Arabidopsis*. In two-choice assays, two transgenic *Arabidopsis 35S*:*YFP-NSs* (*NSs-1*; *NSs-2*) lines were significantly more attractive to thrips compared to controls (Fig 5D), supporting the conclusion that NSs protein can modify vector feeding behavior in a terpene-dependent manner.

### NSs promotes thrips vector performance by targeting MYCs

**Fig 6:**
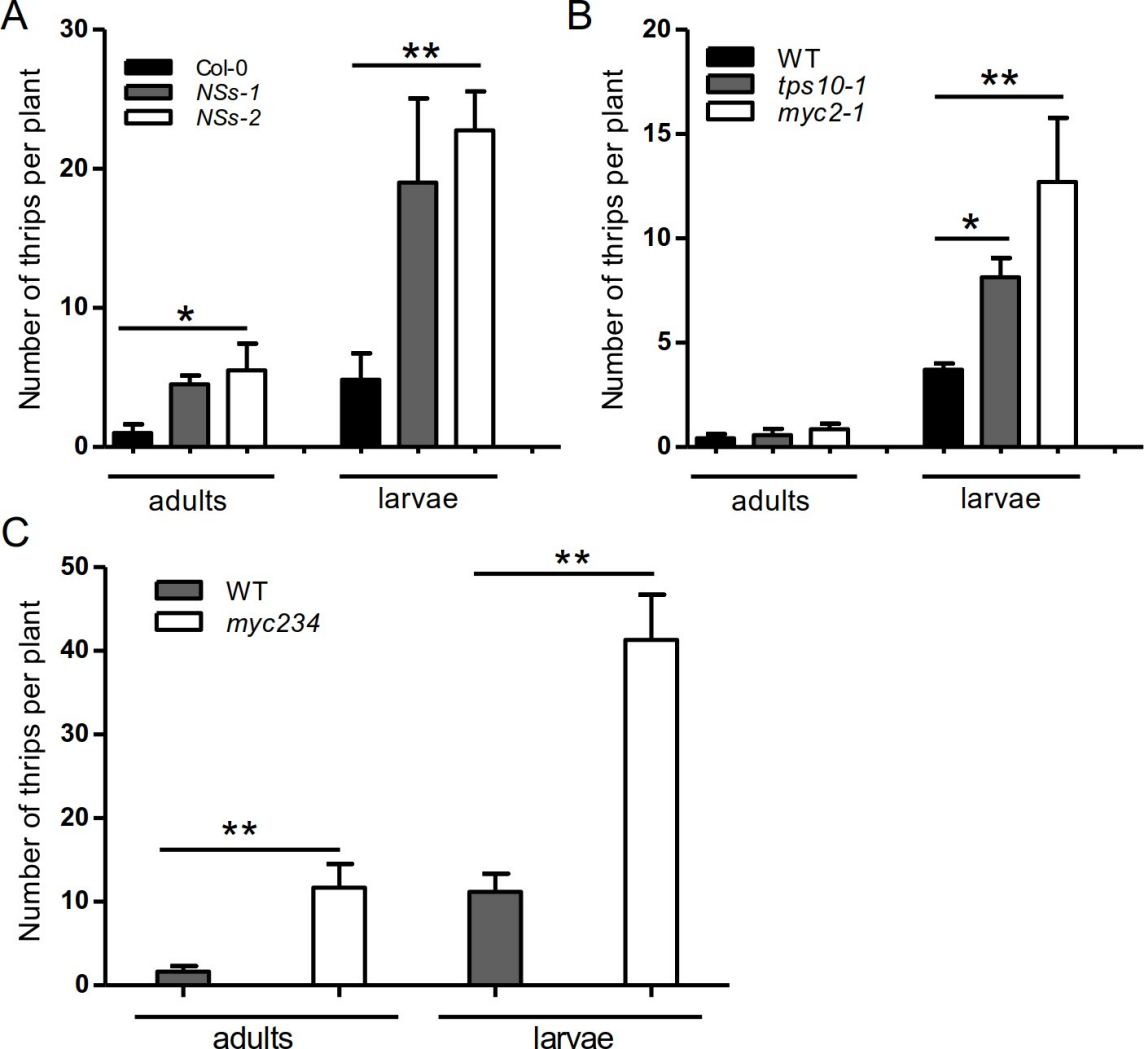
NSs promotes WFT performance by targeting MYC-mediated host defense. (A-C) Effects of different genes on the number of WFT offspring. Seven adult females fed on each three-week-old *Arabidopsis* line. After 2 weeks, new larvae and adults were counted. Values are means ± SE, n = 8. *P < 0.05, **P < 0.01, Student’s *t*-test.

The viral transmission cycle can be roughly divided into two phases. In the first phase, the TSWV-infected plants attract non-viruliferous thrips to feed, with volatiles playing a key role in this early process (Figs 1–5). In the second phase, a (viruliferous) thrips population is established on TSWV-infected plants to facilitate virus transmission. To investigate whether NSs influences thrips population establishment, we performed a thrips spawning experiment with a slight modification [40]. Seven female adult thrips were allowed to feed on *35S*:*YFP-NSs* (*NSs-1; NSs-2*) or wild-type *Arabidopsis* for two weeks. We counted the number of new adults and larvae to analyze the effect of NSs on the thrips population. Plants expressing *NSs* were more suitable for WFT population growth than wild type (Fig 6A). We reasoned that NSs targets MYCs to disable the activation of *terpene synthase* genes, thereby attenuating the defense of the host plant against thrips. To investigate this hypothesis, we conducted another spawning experiment using *myc2-1*, *tps10-1*, and *myc234* mutants. More WFT were found on the mutants compared with wild type; these lines were equally suitable for WFT growth compared to the lines expressing *NSs*, confirming the important role for NSs in the tripartite WFT–TSWV–plant interaction (Fig 6B and 6C).

### A conserved protein interaction between *Orthotospovirus* NSs and plant MYC2

**Fig 7:**
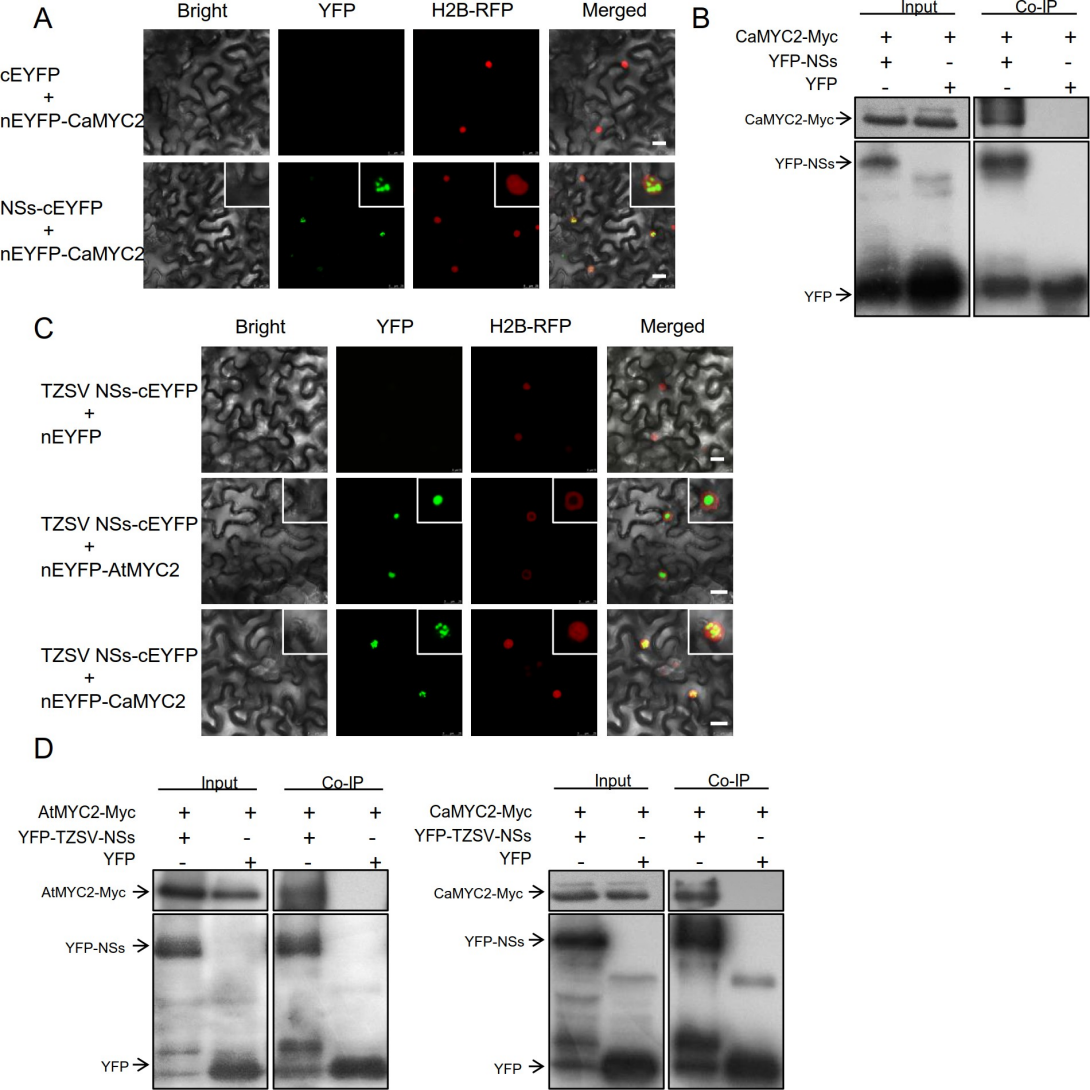
A conserved protein interaction between *Orthotospovirus* NSs and plant MYC2 proteins. (A) BiFC assays of the interaction between TSWV NSs and CaMYC2. H2B-RFP transgenic *N*. *benthamiana* plants, which express a nucleus marker were used in this assay. Bars = 15 μm. (B) Co-immunoprecipitation (Co-IP) assay of the interaction between TSWV NSs and CaMYC2. GFP-trap beads were used to precipitate the interaction complex. (C) BiFC assays of the interaction between TZSV NSs–AtMYC2 and TZSV NSs–CaMYC2. H2B-RFP transgenic *N*. *benthamiana* plants were used in this assay. Bars = 15 μm. (D) Co-immunoprecipitation (Co-IP) assay of the interaction between TZSV NSs–AtMYC2 (left panel) and TZSV NSs–CaMYC2 (right panel). GFP-trap beads were used to precipitate the interaction complex.

TSWV-infected pepper plants were more attractive to the thrips vector than healthy plants (Fig 1A). Therefore, we asked whether NSs could interact with AtMYC2 orthologs in pepper. We examined the interaction between NSs and the homologous protein of AtMYC2 in pepper (CaMYC2). Our BiFC assay results showed interaction fluorescence of NSs–CaMYC2 in the nucleus, while there was no fluorescence of control (Fig 7A). In Co-IP assays, CaMYC2-Myc protein was coimmunoprecipitated by YFP-NSs, but not by YFP alone (Fig 7B). Taken together, our results suggest that NSs–MYC2 interaction is relatively conserved in pepper.

Other orthotospoviruses also encode a NSs protein and might similarly manipulate vector behavior to accelerate their own transmission [49]. To explore whether the interaction between NSs–MYC2 is conserved among orthotospoviruses, we used Tomato zonate spot orthotospovirus (TZSV), a new species of genus *Orthotospovirus* that threatens food security in Southwest China [50]. The evolutionary relationship of TSWV and TZSV is not very close [51], as TSWV represents the American- and TZSV represents the Euro/Asian-type orthotospoviruses (S4 Fig). We examined TZSV NSs–AtMYC2/CaMYC2 interactions by BiFC and Co-IP assays. Notably, BiFC showed interaction fluorescence of TZSV NSs–AtMYC2 and TZSV NSs–CaMYC2 aggregated in the nucleus, while the Co-IP assays again confirmed the interaction between TZSV NSs–AtMYC2 (left panel) and TZSV NSs–CaMYC2 (right panel) (Fig 7D), providing evidence that TZSV NSs interacts with both AtMYC2 and CaMYC2 *in vivo*, consistent with NSs–MYC2 interaction in TSWV (Figs 4 and 7A). These results indicated that the interaction between NSs and MYC2 may be conserved in orthotospoviruses.

In summary, our results suggest that NSs targets MYCs to attenuate host defense responses to thrips, thereby manipulating terpene-dependent chemical communication between the plant and the thrips vector.

## Discussion

### TSWV suppresses host terpene biosynthesis and promotes the performance of its thrips vector

Vector-borne virus-infected plants often attract the pathogens’ vectors [1]. Here, we demonstrate a possible molecular mechanism of this virus-induced indirect manipulation through the shared host plant. Non-viruliferous thrips feeding was reported to induce a negative change in plant quality for their survival [10]. Consistent with this, we showed that the expression of various *TPSs* were induced strongly by herbivory (Fig 1B) and repellent terpenes were produced as a consequence (S2 Fig). Orthotospoviruses depend on the vector thrips for transmission, and enhanced performance of WFT on virus-infected plants would be beneficial to the virus and the vector. We found that the induction of plant defense was greatly decreased in TSWV-infected plants, thus promoting the performance of WFT vector (Figs 1, 5 and 6). Our results establish the existence of an indirect mutualistic relationship between Orthotospoviruses and the thrips vector. This indirect mutualism refers to a positive effect of virus on its insect vector. Virus suppresses plant defense against the insect vector leading to enhanced vector performance and population, which in turn promote virus transmission.

Among the monoterpenes manipulated by TSWV in various plants, linalool functions as a repellent to WFT both in pepper and *N*. *benthamiana* (Fig 2). It is one of the most common defensive monoterpene compounds in the HIPVs released from plant species in response to herbivore attacks [52]. Linalool has been shown to affect the feeding behavior of insects, as well as to attract pollinators, repel herbivores, and affect insect spawning decisions [38,52]. It also inhibits the growth of WFT [53], in agreement with the conclusion that linalool is an anti-WFT secondary metabolite hijacked by TSWV (Fig 2A). Since volatiles are essential to herbivory responses, exogenous application of monoterpenes such as linalool may be a promising approach to avoid herbivore feeding damage and even plant pathogen transmission under field conditions, without the need for engineering in plants.

### NSs represses MYC2-mediated JA signaling pathway to achieve indirect tospovirus–WFT mutualism

Behavioral manipulation has been observed in animal-infecting bunyaviruses for many years. As early as 1980, *La Crosse virus* (LACV) was reported to modify the feeding behavior of mosquito vectors [18]. *Rift Valley fever virus* (RVFV) was found to affect mosquito vector morbidity and mortality [19]. However, the molecular mechanism underlying this manipulation was unclear, and no specific information was available regarding viral determinants of the virus–host–vector interaction in bunyaviruses.

Our study identifies NSs of TSWV as an indirect vector behavior manipulator that suppresses host plant defense responses to attract and benefit the fitness of WFT, which in turn facilitates disease dispersal from plant to plant. Notably, NSs is conserved in bunyaviruses, and TSWV NSs is an avirulence determinant that triggers a hypersensitive response in resistant plants [54]. NSs is also a well-known viral suppressor of host RNA interference in both plants and insects and is essential for TSWV transmission by WFT [16,23–26]. Here, we showed that the expression of *NSs* is sufficient to control the behavior of WFT (Figs 3, 5D and 6A) by suppressing the host defense against insects through MYC proteins (Fig 4). Additionally, the non-viruliferous female thrips were reported to produce more offspring on virus-infected plants, which is in agreement with their preference for TSWV-infected plants [9,10,21]. Taken together, the infection of TSWV could counter plant defense to benefit its vector, thus promoting its spread through the NSs protein.

### Effectors target the plant MYC immunity hub

Earlier studies showed that effectors from bacterial, fungal and oomycete pathogens converge onto common host proteins in *Arabidopsis* [55]. Our results suggest that viral effectors also share the same plant targets. JA signaling is essential for plant defense against pathogen and insect attack in several phytopathological systems [56,57]. However, plant arboviruses target JA signaling to increase the suitability of host plants for their vectors [38,58]. JA-dependent plant defenses affect WFT performance and preference, and TSWV infection reduces the levels of these responses. In JA-insensitive *coi1-1* mutants, WFT do not show a preference for TSWV-infected plants [21]. Our results suggest that the MYC proteins involved in the JA pathway are responsible for plant terpene immunity against WFT (Fig 5A–5C). *MYCs* are downstream genes of the JA receptor COI1, and MYC2-orchestrated transcriptional reprogramming occurs during JA signaling [48].

Functional blocking of *MYCs* increases WFT preference and promotes WFT performance, including developmental duration and fecundity in *Arabidopsis* (Fig 6). We hypothesize that several MYC-regulated indole and aliphatic glucosinolates that function as defensive chemicals against herbivores might be repressed. Alternatively, the levels of nutrients (such as amino acids) are likely altered in the host, which could affect the feeding behavior and preference of thrips, as previously reported [8]. In addition, the interaction between TZSV NSs and MYC2 indicates that TZSV infection of plants may also benefit its insect vector like TSWV infection does (Fig 7B). Therefore it seems like NSs of *Orthotospovirus* conservatively interacted with MYC2 and its homologs in plant host (Fig 7).

By interrupting MYC-regulated plant defense via NSs, *Orthotospovirus* species appear to indirectly manipulate the preference and performance of WFT, as is the case for βC1 in *Begomovirus*. We previously demonstrated that βC1 of *Tomato yellow leaf curl China virus* (TYLCCNV) interacts with MYC2 to subvert plant resistance and to promote vector performance [38]. Notably, *Begomovirus* and *Orthotospovirus* species are persistently transmitted, which tend to induce attraction and promote performance of vectors on infected plants for increased transmission efficiency, indicating that viruses with same transmission mechanisms can have common manipulation tactics. Interestingly, the silencing suppressor 2b of the nonpersistently transmitted virus *Cucumber mosaic virus* (CMV, *Bromoviridae*) also suppresses JA signaling, and *myc234* triple mutant plants were observed to attract the CMV aphid vector [58], although CMV appears to attract vectors deceptively [15]. These similar results on evolutionarily different viruses and plant hosts suggest that manipulation of the JA pathway could be a general feature in tripartite virus–vector–plant interactions. Notably, these independently evolved virulence proteins were known as silencing suppressors that convergently targeted the host RNA silencing machinery, and our studies establish that the same occurs for the manipulation of plant–insect vector interactions.

These similar effects and pathogen manipulation tactics indicate that the mechanistic and evolutionary principle for diverse pathogens seems to be convergent, even in human pathogens. For instance, CCR5, which is the first described cellular receptor of human immunodeficiency virus (HIV), is necessary and sufficient for the pathogenesis of many pathogens [59]. The HIV, *Toxoplasma gondii*, poxviruses (vaccinia and myxoma), and *Staphylococcus aureus* exploit CCR5 to target and kill mammalian immune cells [60–63]. Why pathogens from different kingdoms tend to keep finding the same host targets to disrupt their defenses, and whether this is a consequence of selective pressure in evolution remain to be further determined.

In summary, we have demonstrated that the emission of several monoterpenes is greatly decreased by the TSWV infection, which in turn promotes WFT preference and performance, uncovering a molecular mechanism underpinning the virus-induced manipulation through the shared host plant of the WFT vector. This work presents a mechanism by which a pathogen regulates host-derived olfactory cues for vector attraction. These results will also help to address similar tripartite interaction systems in plants, animals and humans and will allow innovative control methods through interference of vector transmission.

## Materials and methods

### Plant materials

Pepper accession Lingfeng (*Capsicum annuum* L.), *Nicotiana benthamiana* and *Arabidopsis thaliana* (Col-0) plants were grown in insect-free growth chambers following standard procedures [38]. The *Arabidopsis myc2-1*, *tps10-1*, and *myc234* mutants (Col-0 background) were described previously [38]. The *35S*:*YFP-NSs* transgenic lines *NSs-1* and *NSs-2* were generated using the *Agrobacterium*-mediated floral-dip method [64].

### Naive Western flower thrips colony and mechanical inoculation of TSWV

A starting colony of Western flower thrips (WFT, *Frankliniella occidentalis* Pergande) (Thysanoptera: Thripidae) was kindly provided by Prof. Youjun Zhang (Institute of Vegetables and Flowers, Chinese Academy of Agricultural Sciences). The thrips were maintained on green bean pods (*Phaseolus vulgaris* L.) in a climate chamber as described previously [65]. Tomato spotted wilt orthotospovirus (isolate TSWV-YN) obtained from Prof. Xiaorong Tao (Nanjing Agriculture University) was mechanically inoculated onto pepper and *N*. *benthamiana* as described by Mandal et al. [66]. Infected leaves were ground in 0.05 M phosphate buffer (pH 7.0) and applied to the host plant using a soft finger-rubbing technique. Infected plants were tested at 10–14 dpi by RT-qPCR prior to the thrip two-choice assays.

### Thrip two-choice assay

The two-choice assays on plants or leaves were performed as described previously [8,9]. Peppers inoculated with TSWV or buffer was used for the assay at 10–14 days post inoculation. A TSWV-infested and a control plant were confined in a pot covered with a fine mesh. For *N*. *benthamiana*, detached leaves of TSWV-infected plants and non-infected plants were separately placed in a 16 cm-Petri dish, which was covered with a moist filter paper. For *Arabidopsis*, plants were cultivated on solid Murashige and Skoog medium for 3–5 weeks, and whole plants were used for the two-choice assay. Fifty *F*. *occidentalis* adults were released to the center of the two tested plants or the leaves of *N*. *benthamiana*, the number of thrips that settled on each plant or leaf was counted at 12h (pepper) or 24 h (*N*. *benthamiana*, *Arabidopsis*) after release. For two-choice assays with individual monoterpene, 2 cm × 2 cm filter paper containing 40 μL of a 1:100 (v/v) solution of standard chemical substance from Sigma dissolved in n-hexane or n-hexane alone (as a control) was placed in a 16cm-Petri dish. Thrips were released between the two tested samples, and the thrips were counted 5 min after release. The Petri dishes were contained in a thrip culture chamber throughout the experiment to maintain consistent environmental conditions.

### Thrip infestation assay

Plants were infested with non-viruliferous thrips as described previously [56]. Twenty adult thrips (7–14 d after eclosion) were grouped and starved for 3 h before the plant infestation assay. *Arabidopsis* plants grown on solid MS medium or soil-grown pepper and *N*. *benthamiana* plants were infested with adult thrips for the indicated time period. The thrips were gently removed and the leaf samples collected in liquid nitrogen for further analysis. For the *GUS*-reporter line expression assays, transgenic *Arabidopsis* plants were infested with thrips for 24 h, followed by GUS activity analysis. The experiment was repeated at least twice with similar results.

### Volatile analysis

For volatile analysis on pepper plants, plants were infested with thirty adults in a nylon mesh cage for 6 h before volatile collection. The volatiles emitted from insect‐exposed TSWV-infected and control plants were collected with a solid phase microextraction (SPME; Supelco, Belafonte, PA, USA) fiber consisting of 100 μm polydimethylsiloxane (Supelco). Chemical analysis was performed by gas chromatography-mass spectrometry (GC-MS) (Shimadzu, QP2010) coupled with a DB5MS column (Agilent, Santa Clara, CA, USA, 30 m x 0.25 mm x 0.25 μm). The SPME fiber was thermally desorbed in the injector at 250°C for 1 min. The initial oven temperature was held at 40°C for 3 min, increased to 240°C with a gradient of 5°C/min, and maintained at 240°C for 5 min. The inlet temperature was 250°C. The collection of volatiles for each treatment was repeated 4–6 times.

The collection, isolation, and identification of volatiles from *N*. *benthamiana* plants were performed as described previously [38,67]. Plant volatiles were collected for 12 h at a gas flow rate of 300 mL/min and analyzed by GC-MS. At least four plants per group were used.

### Plasmid construction

For PVX heterologous virus protein expression in pepper, the TSWV virus genes *NSs*, *NSm*, and *Ncp* were cloned into the PVX vector pGR208 by using gene-specific primers in S1 Table. For agroinfiltration transient expression vectors construction, the indicated DNA fragments were PCR cloning into pENTR-3C entry vector, then transformed into the agroinfiltration destination vector under the control of a *CaMV 35S* promoter. All constructs used for protein expression in plants were transformed into *Agrobacterium tumefaciens* strain EHA105. *Agrobacterium* carrying the binary vectors were infiltrated into the abaxial sides of pepper and *N*. *benthamiana* leaves [49].

### Yeast two-hybrid analysis

The *Arabidopsis* Mate and Plate Library was screened using yeast mating method according to the Matchmaker Gold Yeast Two-Hybrid System manufacturer’s protocol (Clontech). Briefly, full-length *NSs* was amplified and inserted into the pGBKT7 vector by Gateway recombination, then the constructs was transformed into yeast strain Y2HGold and testing for autoactivation by using the Yeastmaker Yeast Transformation System (Clontech). Then the *Arabidopsis* Mate and Plate Library and BD-NSs yeast clones were mated in YPDA medium. After incubation, isolated destination clones were selected from diploid-selection medium (SD/-Leu/-Trp). These primary positive interactors were secondary screened on medium plates (SD/-Leu/-Trp/-His) and third time screened on medium plates (SD/-Leu/-Trp/ -His/X-a-Gal). PCR and BLAST searches were used to obtain sequence information on corresponding AD- and BD-clones per colony.

The interaction between TSWV NSs and AtMYCs were confirmed according to the manufacturer’s protocol (Clontech). The pGBKT7-NSs and pGAD424-MYCs constructs were co-transformed into yeast strain Y2HGold. Yeast cotransformed with the indicated plasmids was spotted onto synthetic medium (SD-Leu-Trp-His) containing 10 mM 3-amino-1,2,4-triazole and 0.04 mg/mL X-α-gal. The empty vectors pGBKT7 (BD) and pGADT7 (AD) were used as negative controls [38].

### Bimolecular fluorescence complementation (BiFC)

BiFC was performed as described previously [38]. The indicated constructs were fused with the N-or C- terminal of YFP and transformed into *A*. *tumefaciens* strain EHA105. The recombinant constructs of *A*. *tumefaciens* were infiltrated in 4–6 week old transgenic *N*. *benthamiana* (expressing a nuclear marker-H2B-RFP)[68] leaves via agroinfiltration. The fluorescent signals were detected at 2 dpi via confocal microscopy.

### *In Vitro* pull-down assay

His and GST tag fusion proteins were purified using His- and GST-Trap (GE Healthcare) according to the manufacturer’s instructions [38]. GST-AtMYC2 (2 μg) and His-NSs (2 μg) fusion proteins were mixed and incubated with 25 μL GST-Trap for 2 h at 4°C in a binding buffer (50 mM Tris-HCl, pH 7.5, 200 mM NaCl, 0.25% Triton X-100, and 35 mM b-mercaptoethanol). After six washes with binding buffer, pulled-down proteins were resuspended in 2xSDS buffer and detected by immunoblot using Anti-GST and Anti-His antibodys.

### Co-immunoprecipitation (Co-IP)

*A*.*tumefaciens* carrying the *35S*:*MYC2-Myc* or *35S*:*YFP-NSs* constructs were infiltrated into *N*. *benthamiana* leaves. About 1g leaf tissue was collected and ground to powder in liquid nitrogen. Proteins were extracted in a cold extraction buffer (50 mM Tris-HCl, pH 7.5, 150 mM NaCl, 2 mM MgCl2, 0.5 mM EDTA, 0.1% Triton, 0.5% NP-40, 10% glycerol, 1 mM phenylmethylsulfonyl fluoride (PMSF), one protease inhibitor cocktail/100 mL (Sigma-Aldrich, USA)). Then the protein extracts were incubated with 25 μL GFP-trap beads for 3 h at 4°C. After that, the beads were washed three times with extraction buffer and resuspended in 2xSDS buffer before used for immunoblot analysis.

### Quantitative RT-PCR

Total RNA was extracted from leaf and plant samples using an RNeasy Plant Mini Kit (Qiagen) with column DNase treatment. RNA was reverse transcribed using TransScript One-Step gDNA Removal and cDNA Synthesis SuperMix (TransGen Biotech, China). Four to six independent biological samples were collected and analyzed. RT-qPCR was performed using SYBR Green Real-Time PCR Master Mix (Toyobo, China) on the CFX 96 system (Bio-Rad). Pepper *Ca-ACT1* and *N*. *benthamiana Nb-EF1α* were used as the internal controls (Listed in S1 Table).

### Thrip spawning assay

The thrip spawning assay was performed as described previously with some modifications [40]. *Arabidopsis* plants were grown in soil covered with Parafilm (Bemis, USA) to prevent any thrips from escaping and to facilitate counting. Three-week-old plants were placed in an acryl cylinder chamber (7 cm × 5 cm) and covered with a fine mesh. Seven female adults (7–14 d after eclosion) were allowed to infest a single plant for two weeks, and new larvae and adult thrips were counted. Eight plants of each genotype were used per experiment. The experiment was repeated at least twice with similar results.

### GUS staining

Transgenic *Arabidopsis* plants expressing the *AtMYC2* or *AtTPS10* promoter:*GUS* reporter gene were infested with thrips for 24 h and incubated in GUS staining buffer (0.5 mg/mL X-glucuronide, 0.5 mM potassium ferricyanide, 0.5 mM potassium ferrocyanide, 10 mM EDTA, 0.1% Triton X-100, 0.1 M pH 7.0 phosphate buffer) at 37°C overnight. The stained seedlings were cleared by washing with 70% ethanol. Untreated plants were used as a negative control. The experiment was repeated at least twice with similar results.

### Data analysis

Significant differences in gene expression and volatile organic compound levels were determined by Student’s *t* tests or one-way ANOVA; if the ANOVA result was significant (P < 0.05), Duncan’s multiple range tests were used to detect significant differences between groups. Thrip choices between different treatments were analyzed by nonparametric Wilcoxon matched pairs tests. All statistical tests were carried out with GraphPad Prism.

### Accession numbers

Sequence data in this study can be found in Sol Genomics Network (https://solgenomics.net), TAIR (www.Arabidopsis.org) or GenBank/EMBL under the following accession numbers: CaMYC2 (CA00g50270), CaMTS1 (CA08g16370), CaMTS2 (CA08g16380), CaMTS3 (CA08g16410), CaMTS4 (CA08g16420), AtMYC2 (AT1G32640), AtMYC3 (AT5G46760), AtMYC4 (AT4G17880), AtTPS10 (AT2G24210), TSWV NSs (JF960235.1), TSWV NSm (JF960236.1), and TSWV Ncp (JF960235.1), TZSV(EF552433.1).

## Supporting information

S1 Fig MeJA induces several *TPS* genes expression in *N. benthamiana* similar to thrips infestation. (A) Relative expression levels of various *TPS* genes in *N*. *benthamiana* after thrips infestation. Four-week-old *N*. *benthamiana* plants were infested with twenty thrips adults in a confined pot for 48h. Total RNA was prepared from treated plants for RT-qPCR analysis. Values are means + SE, n = 3. **P < 0.01, Student’s *t*-test. (B) Relative expression levels of various *TPS* genes in *N*. *benthamiana* after 100 μM MeJA treatment for 24h. Values are means + SE, n = 3. **P < 0.01, Student’s *t*-test.
(TIF)

S2 Fig Thrips infestation induced plant volatiles emission in peppers. Representative extracted ion chromatograms of GC/MS headspace volatile compounds of peppers. Plants under the same growth condition were infested with (pepper-thrips) or without (pepper) thrips for 6 h.
(TIF)

S3 Fig TSWV NSs interacts with AtMYC3 and AtMYC4. (A) Interaction between TSWV NSs–AtMYC3 and TSWV NSs–AtMYC4 in a yeast two-hybrid assay. Yeast cotransformed with the indicated plasmids was spotted onto synthetic medium (SD-Leu-Trp-His) containing 0.04 mg/mL X-α-gal and 10mM 3-amino-1,2,4-triazole (3-AT). The empty vectors pGBKT7 (BD) and pGADT7 (AD) were used as negative controls. (B) Interaction between TSWV NSs–AtMYC3 and TSWV NSs–AtMYC4 in a BiFC assay. Indicated construsts were transiently expressed in H2B-RFP transgenic *N*. *benthamiana* leaf epidermal cells by agroinfiltration. Bars = 15 μm.
(TIF)

S4 Fig Phylogenetic tree of NSs protein from diverse orthotospoviruses. ClustalW was used to construct the phylogenetic tree. It was constructed based on the amino acid sequences of the NSs protein from 23 orthotospoviruses.
(TIF)

S1 Table DNA primers used in this study. (DOCX)
