# Supplementary figures and images for: The Orthotospovirus nonstructural protein NSs suppresses plant MYC-regulated jasmonate signaling leading to enhanced vector attraction and performance
Source: PLoS Pathog. 2019 Jun 17;15(6):e1007897. doi: 10.1371/journal.ppat.1007897 (PMC6598649; doi:10.1371/journal.ppat.1007897)

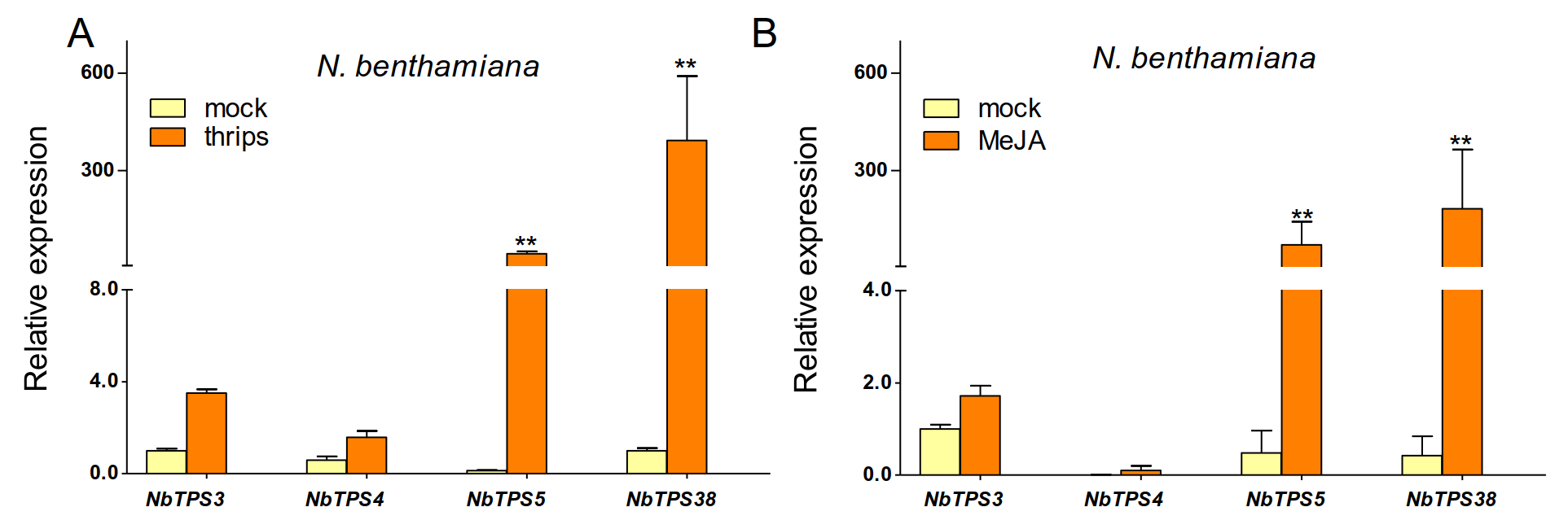

Supplement: S1 Fig — (A) Relative expression levels of various TPS genes in N. benthamiana after thrips infestation. Four-week-old N. benthamiana plants were infested with twenty thrips adults in a confined pot for 48h. Total RNA was prepared from treated plants for RT-qPCR analysis. Values are means + SE, n = 3. **P < 0.01, Student’s t-test. (B) Relative expression levels of various TPS genes in N. benthamiana after 100 μM MeJA treatment for 24h. Values are means + SE, n = 3. **P < 0.01, Student’s t-test. (TIF) [file ppat.1007897.s001.tif]

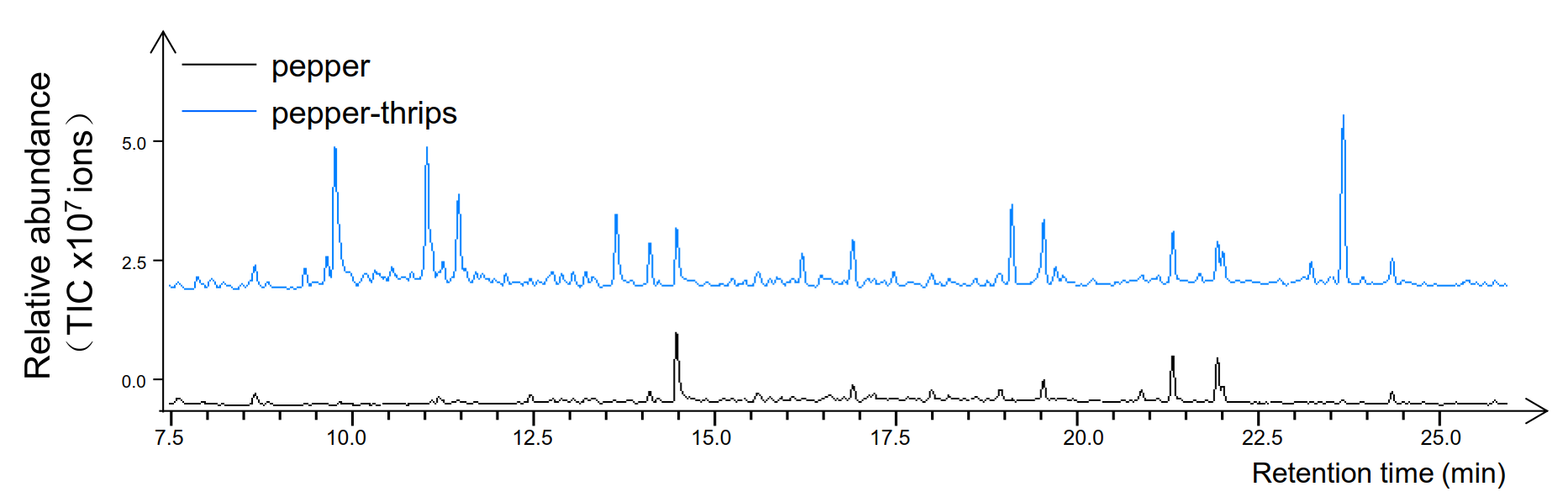

Supplement: S2 Fig — Representative extracted ion chromatograms of GC/MS headspace volatile compounds of peppers. Plants under the same growth condition were infested with (pepper-thrips) or without (pepper) thrips for 6 h. (TIF) [file ppat.1007897.s002.tif]

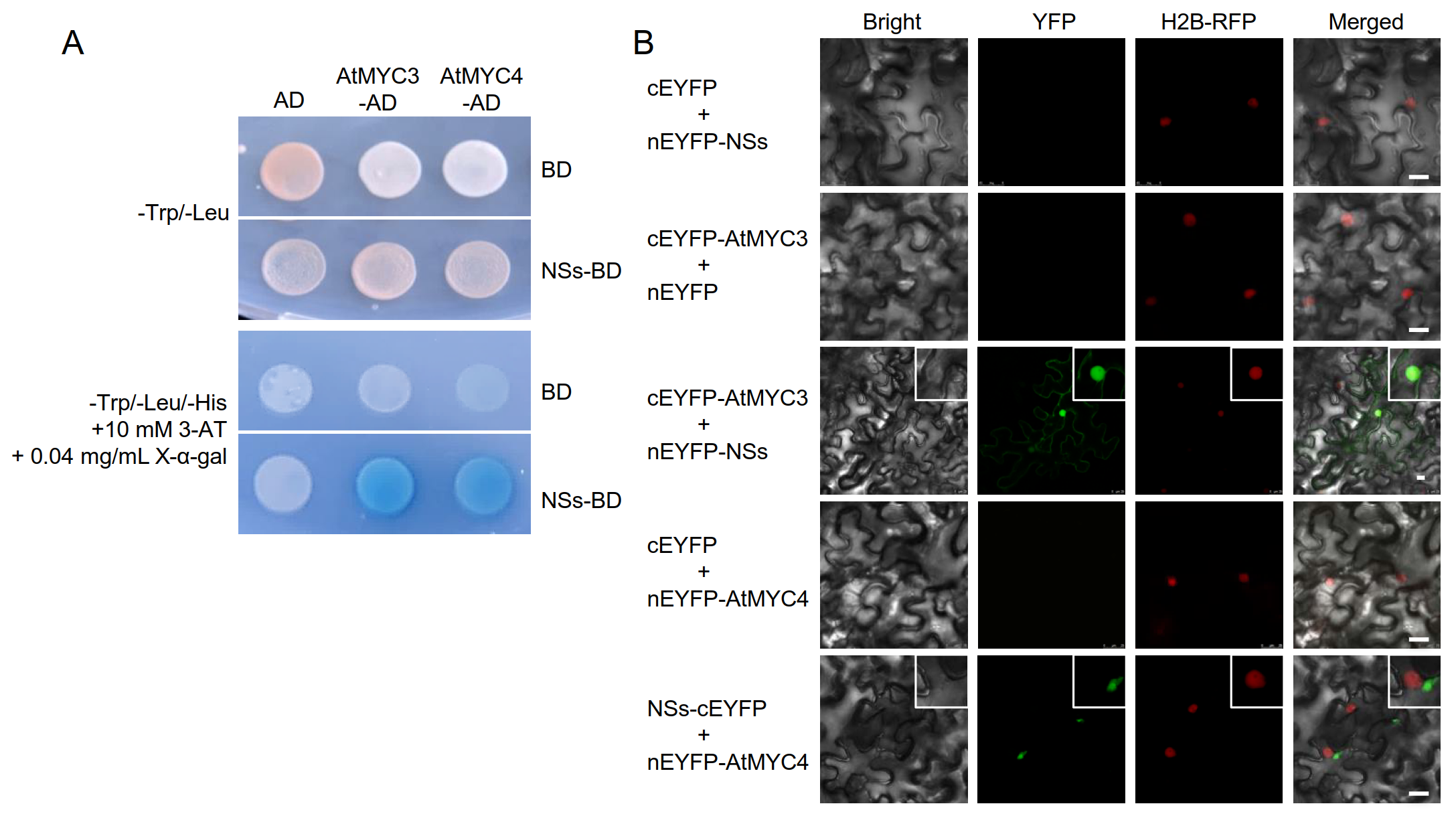

Supplement: S3 Fig — (A) Interaction between TSWV NSs–AtMYC3 and TSWV NSs–AtMYC4 in a yeast two-hybrid assay. Yeast cotransformed with the indicated plasmids was spotted onto synthetic medium (SD-Leu-Trp-His) containing 0.04 mg/mL X-α-gal and 10mM 3-amino-1,2,4-triazole (3-AT). The empty vectors pGBKT7 (BD) and pGADT7 (AD) were used as negative controls. (B) Interaction between TSWV NSs–AtMYC3 and TSWV NSs–AtMYC4 in a BiFC assay. Indicated construsts were transiently expressed in H2B-RFP transgenic N. benthamiana leaf epidermal cells by agroinfiltration. Bars = 15 μm. (TIF) [file ppat.1007897.s003.tif]

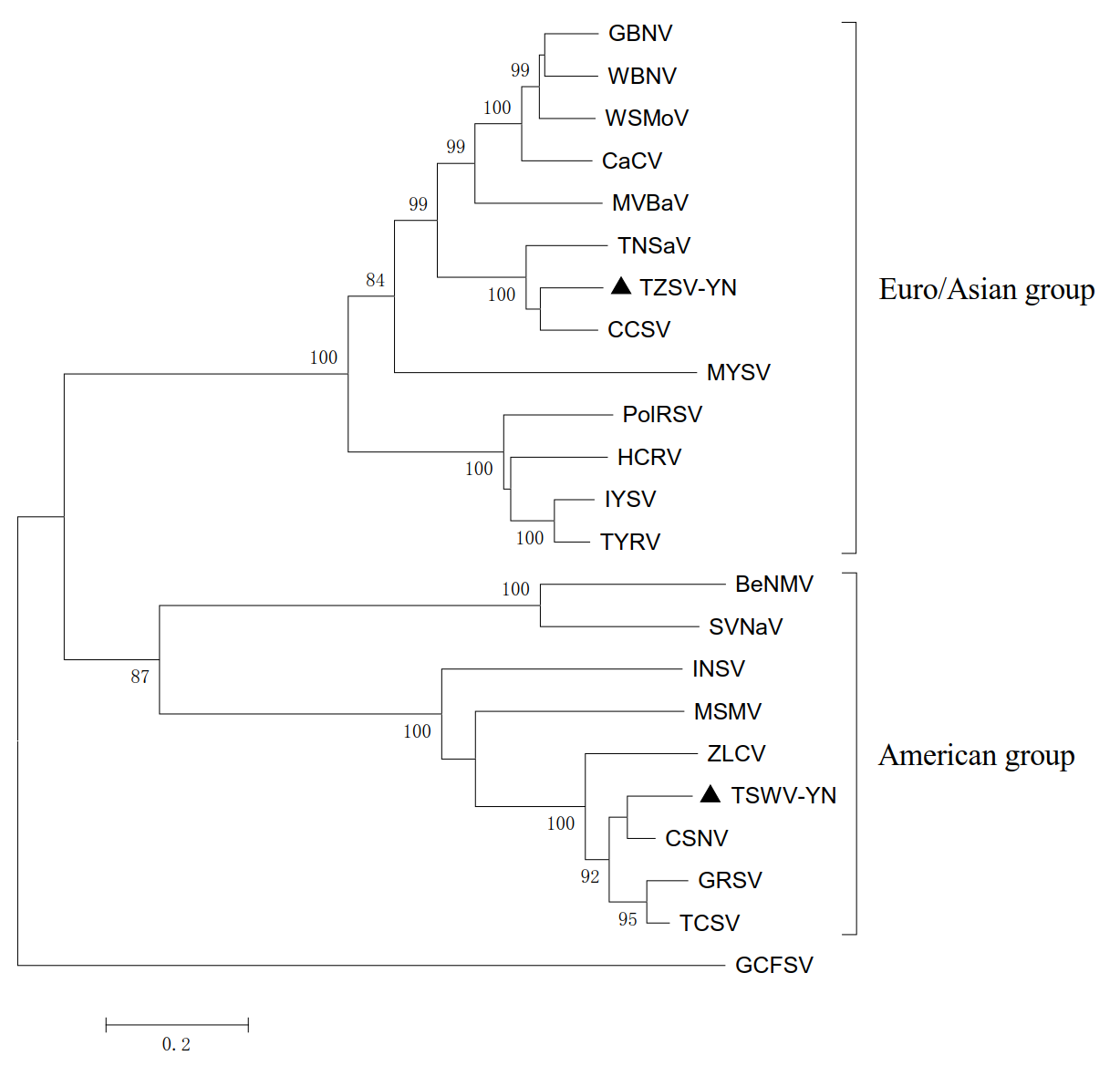

Supplement: S4 Fig — ClustalW was used to construct the phylogenetic tree. It was constructed based on the amino acid sequences of the NSs protein from 23 orthotospoviruses. (TIF) [file ppat.1007897.s004.tif]
