# Supplementary material for: The Orthotospovirus nonstructural protein NSs suppresses plant MYC-regulated jasmonate signaling leading to enhanced vector attraction and performance
Source: PLoS Pathog. 2019 Jun 17;15(6):e1007897. doi: 10.1371/journal.ppat.1007897 (PMC6598649; doi:10.1371/journal.ppat.1007897)
Supplement: S1 Table — (DOCX) [file ppat.1007897.s005.docx]

**S1 Table.** **DNA primers used in this study.**

| Gene | Sequence (5’-3’) | Purpose |
| --- | --- | --- |
| Ca-ACT1-F | GACGTGACCTAACTGATAACCTGAT | RT-qPCR |
| Ca-ACT1-R | CTCTCAGCACCAATGGTAATAACTT | RT-qPCR |
| Ca-MST1-F | GAAGAGTGTTTCTGAAGAAGTAGCA | RT-qPCR |
| Ca-MST1-R | TTATACAACTGAAATTGTGATGGG | RT-qPCR |
| Ca-MST2-F | GTCTTTCTACAACCACTAGAAGTGAC | RT-qPCR |
| Ca-MST2-R | CATCATCAAATTCTTCTTGATTTC | RT-qPCR |
| Ca-MST3-F | ATGCTATCCATGGCCACCTCAAGG | RT-qPCR |
| Ca-MST3-R | ATCGCTCCCTATACTTCTCTCC | RT-qPCR |
| Ca-MST4-F | CATGGGTCTCAATTGCGGTA | RT-qPCR |
| Ca-MST4-R | TCTAACGTGTTCTCTTGCCTC | RT-qPCR |
| Nb-EF1α-F | TGGTGTCCTCAAGCCTGGTATGGTTG | RT-qPCR |
| Nb-EF1α-R | ACGCTTGAGATCCTTAACCGCAACATTCTT | RT-qPCR |
| Nb-TPS3-F | TCACCAACCCAATTACGAAAGAGA | RT-qPCR |
| Nb-TPS3-R | CGTTTATCATTTTCCATGTCTCCT | RT-qPCR |
| Nb-TPS4-F | CGGATGAATTGAAGAGGGGTGATGTT | RT-qPCR |
| Nb-TPS4-R | ATGTGCTGTTCTTGCAATATTCTTT | RT-qPCR |
| NbTPS5-F | ACATTGTTCAAGCAACACATCAAGAA | RT-qPCR |
| NbTPS5-R | CATCAAGAGTTGTAACAAGAGCATT | RT-qPCR |
| NbTPS38-F | ACGCAATAGAGCGACTACCTGACTA | RT-qPCR |
| NbTPS38-R | TGTTTGTTATCCATGCATTTCTCA | RT-qPCR |
| n-At-MYC2-F | GGCAGCATATGATGACTGATTACCGGCTAC | Gene cloning |
| n-At-MYC2-R | GACTGCCCGGGTTAACCGATTTTTGAAATC | Gene cloning |
| n-At-MYC3-F | GGTACCATGAACGGCACAACATCA | Gene cloning |
| n-At-MYC3-R | CTCGAGCAATAGTTTTCTCCGAC | Gene cloning |
| n-At-MYC4-F | GGTACCATGTCTCCGACGAATGTT | Gene cloning |
| n-At-MYC4-R | CTCGAGCATGGACATTCTCCAAC | Gene cloning |
| n-Ca-MYC2-F | GGTACC ATGAATATATGGAGTACTAGTAACACC | Gene cloning |
| n-Ca-MYC2-R | GCGGCCGCTCAGCAATTTTCGATG | Gene cloning |
| TSWV-NSs-F | GGTACCATGTCTTCAAGTGTTTATG | Gene cloning |
| TSWV-NSs-R | GCGGCCGCTTTGATCCTGAAGCATAT | Gene cloning |
| TSWV-NSs-PVX-F | ATCGAT ATGTCTTCAAGTGTTTATG | Gene cloning |
| TSWV-NSs-PVX-R | GTCGAC TTTTGATCCTGAAGCATAT | Gene cloning |
| TSWV-Ncp –PVX-F | GCGGCCGC ATGTCTAAGGTTAAGCTC | Gene cloning |
| TSWV-Ncp-PVX-R | GTCGAC AGCAAGTTCTGCAAGTTTT | Gene cloning |
| TSWV-NSm-PVX-F | ATCGAT ATGTTGACTTTTTTTGGT | Gene cloning |
| TSWV-NSm-PVX-R | GTCGAC TATCTCATCAAAAGATAAC | Gene cloning |
| TZSV-NSs-F | GGTACC ATGTCTACTGCAAAGATG | Gene cloning |
| TZSV-NSs-R | CTCGAG GCAGTTTGAACCTTTTC | Gene cloning |
| TSWV-NSs-His-F | GGATCC ATGTCTTCAAGTGTTTATG | Protein expression |
| TSWV-NSs-His-R | CTCGAG TTTTGATCCTGAAGCATAT | Protein expression |
